# Atrial myxoma: a rare cause of hemiplegia in children

**DOI:** 10.5830/CVJA-2016-093

**Published:** 2017

**Authors:** Uchenna Onubogu, Boma West, Boma Orupabo-Oyan

**Affiliations:** Braithwaite Memorial Specialist Hospital, Portharcourt, Rivers, Nigeria; Braithwaite Memorial Specialist Hospital, Portharcourt, Rivers, Nigeria; Braithwaite Memorial Specialist Hospital, Portharcourt, Rivers, Nigeria

**Keywords:** atrial myxoma, hemiplegia, thromboembolic, cardiac, children

## Abstract

**Background:**

Atrial myxoma is an uncommon cause of hemiplegia in children. However hemiplegia is the commonest manifestation of atrial myxoma in the paediatric age group.

**Case report:**

An 11-year-old girl presented with left hemiplegia and palpitations. Three months later she had a deepvein thrombosis of the right common iliac vein. MRI of the brain showed a subacute right thalamic infarct, and an ECG showed left atrial and left ventricular hypertrophy. Transthoracic echocardiography revealed a left atrial myxoma impinging on the mitral valve. A diagnosis of left atrial myxoma with multiple thromboembolic events was made. She was placed on anticoagulants until she died while awaiting surgical tumour resection.

**Conclusion:**

Echocardiography should be done early in children presenting with ischaemic thromboembolic diseases in order to reduce morbidity and mortality rates resulting from cardiac pathology.

## Introduction

Cardiac myxoma is a rare cause of cerebrovascular disease (CVD), especially in children. The common cause of CVD in African children is sickle cell disease.^1^ Other common causes are cyanotic congenital heart diseases, arrhythmias, coagulopathies and systemic infection (meningitis, sepsis).^1^,^2^

The term myxoma is the Latin translation of a Greek word ‘muxa’, which literally means mucus. A cardiac myxoma is a benign tumour of the heart arising from primitive mesenchyme. Cardiac myxoma is the most common primary tumour of the heart in adults but is very infrequent in the paediatric population.^3^ Among primary cardiac tumours in children, the rhabdomyomas are the commonest.^4^

Cardiac myxomas can be seen in any of the cardiac chambers but rarely on the heart valves. The atria are more affected than the ventricles, therefore cardiac myxomas are said to be predominantly intra-atrial. About 90% of cardiac myxomas are located in the atria with a left-to-right ratio of about 4:1.^5^ Our patient had a left atrial cardiac myxoma. The size of a cardiac myxoma can range from small (unnoticeable) to as large as 8 cm in length.

## Case report

An 11-year-old girl was referred to the cardiology clinic on account of left hemiplegia of one month duration. The hemiplegia was of sudden onset and was associated with headache, dizziness and vomiting at onset. She had also been having intermittent episodes of palpitations and had just been discharged from hospital two weeks earlier after being managed for an intracranial space-occupying lesion, with raised intracranial pressure, left hemiplegia and multiple cranial nerve palsy. She had a positive history of sudden death in her family (an uncle and her grandmother).

On examination, she had a hemiplegic gait, a left CN V1, VII palsy and decreased power, tone and reflexes in the left upper and lower limb. A regular pulse and wide blood pressure difference was noted in both right and left upper limbs (right 120/50 mmHg, left 60 mmHg/unrecordable).

Previous tests had been done when she was admitted. MRI of the brain showed subacute right thalamic infarct, and her blood lipid profile and random blood glucose results were normal. Her genotype was AA, mantoux was negative, chest radiograph was normal, and PT/PTTK was also normal.

A diagnosis of peripheral artery disease was entertained and magnetic resonance angiography of both carotid arteries was done, which was normal. The ECG showed sinus rhythm with evidence suggestive of left atrial and left ventricular hypertrophy. An echocardiography could not be done immediately but was requested.

She was subsequently placed on aspirin, encephabol and regular physiotherapy while angiography was being awaited. She defaulted from follow up and was seen in hospital three months later when she collapsed at school after complaining of heaviness of the right side of the body and inability to walk. Her history revealed that she had stopped her aspirin two days earlier.

On examination, she was conscious, and the blood pressure in both upper limbs was equal and normal (110/70 mmHg). Her pulse was a good volume but irregularly irregular, both legs were cold to the touch, and the dorsalis pedis was barely palpable. A diagnosis of deep-vein thrombosis was entertained.

**Fig. 1.**
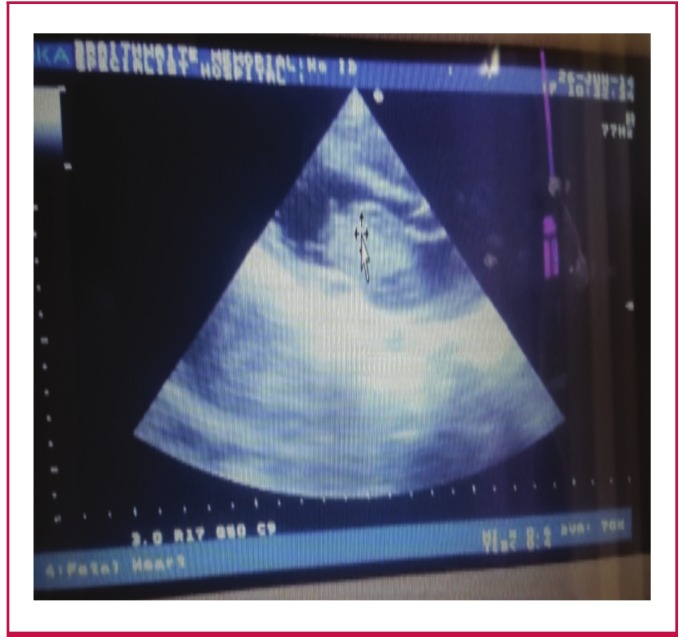
Intra-atrial mass measuring 3.9 x 2.6 cm impinging on the mitral valve, and a dilated left atrium.

She was rehydrated and recommenced on aspirin tablets, while vascular ultrasound of both legs showed a deep focal vein clot in the right common iliac vein. A transthoracic echocardiography was finally done and it revealed a mobile mass in the left atrium (myxoma) measuring 3.9 × 2.6 cm impinging on the mitral valve, a dilated left atrium and multiple ectopic beats (Fig. 1).>

A diagnosis of left atrial myxoma with intermittent arrhythmia and multiple thromboembolic events was made. Anticoagulation was commenced with heparin and warfarin tablets. She was also referred for surgical resection of the atrial myxoma. Due to financial constraints, surgery could not be done and she died 10 months later while awaiting resection of the cardiac tumour.

## Discussion

Cardiac myxoma accounts for 30% of all primary cardiac tumours, with a male-to-female ratio of 2:7.^6^ The mean age of presentation is 56 years for sporadic cases and 25 years for familial cases.^3^,^7^ It is very rarely reported in children; about three case have been reported in Nigerian children.^8^,^9^ A surgical incidence of 0.5 atrial myxomas per million population per year was reported in Ireland.^10^

Myxomas may be associated with several syndromes, namely Carney complex (multiple cardiac and extra-cardiac myxoma, pigmented skin lesions and endocrine hyperactivity), LAMB complex (lentigenosis, atrial myxoma, mucocutaneous myxoma and blue nevi), NAME complex (nevi, atrial myxoma, neurofibromatosis and ephelides-freckles) and a complex with lentigenosis, myxoid fibroma of the breast, skin myxomas and nodular adrenal disease.

Single or multiple gene mutations have been implicated in the aetiology of cardiac myxoma. They are PRKAR1 on chromosomes 17 and 2p16. Autosomal dominant transmission is seen in Carney complex. Atrial myxomas usually occur as a single lesion and rarely as multiple lesions of varying sizes. They may be pedunculated lesions or freely mobile and able to move through the AV valves, as in our patient, or sessile with a broad base.

Cardiac myxomas can be asymptomatic in 20% of cases and present as sudden death in 15% of cases.^11^,^12^ When symptomatic, symptoms may be due to intra-cardiac obstruction to blood inflow and outflow. Our patient’s tumour was causing some obstruction in the mitral valve. Symptoms can also be due to mechanical interference with cardiac function, leading to signs of left- or right-sided heart failure, arrhythmias and syncope, depending on the location. Our patient had syncope and intermittent arrhythmias, which were captured clinically by the irregular pulse; unfortunately when her ECG was done she had converted to normal rhythm.

Patients may also present with symptoms of systemic or pulmonary embolisation due to fragmentation of the tumour cells. Our patient had multiple systemic embolic phenomena affecting the common iliac vein and cerebral vessels. There may also be constitutional symptoms in 50% of patients due to overproduction of interleukin 6 by the tumour cells. These symptoms include fever, weight loss, lightheadedness and arthralgia.

A review of nine paediatric cases with atrial myxoma shows that right hemiparesis was the commonest clinical presentation in the paediatric age group, occurring in eight (89%) of the children. Other common symptoms documented were red spots on the limbs (44%), aphasia (44%), lethargy (22%), seizures, headache, blindness, slurred speech, dizziness and diplopia (11%).^13^ Pridie also described three children with cardiac myxoma; all had systemic emboli involving the central nervous system.^14^

A full blood count and blood film may show normochromic or hypochromic anaemia. They can also have haemolytic anaemia due to mechanical destruction of the erythrocytes by the tumour. Serum interleukin 6 levels may be high and can be used as a marker of recurrence.

Chest radiography can show abnormal cardiac silhouette, mimicking mitral stenosis, tumour calcification and pulmonary oedema. Echocardiography will show an intra-cardiac mass. Transoesophageal echocardiography has better specificity and sensitivity compared to transthoracic echocardiography. We did a transthoracic echocardiograph for our patient. The point of attachment of the tumour is best visualised by MRI or CT scanning. Electrocardiography will show left atrial enlargement, atrial fibrillation, atrial flutter or other conduction disturbances. Our patient had left atrial enlargement.

Molecular genetic testing for PRKAR1 may be positive. Histology shows lipidic cells embedded in a vascular myxoid stroma, polygonal to stellate shaped, with scanty eosinophilic cytoplasm.

Surgical resection is the treatment of choice; open heart or endoscopic resection. Drug therapy is used only for complications such as congestive heart failure or cardiac arrhythmias.

Post-surgery survival is good, however there is an unusual recurrence risk of 1–3% in sporadic cases and 20% in familial cases.^3^,^15^ Biannual echocardiograms are useful for early detection of recurrent tumors post surgery. Relatives of a child with the familial type of atrial myxoma should undergo echocardiography for early detection.

## Conclusion

Atrial myxoma is an uncommon cause of hemiplegia in children. However hemiplegia is the commonest manifestation of atrial myxoma in the paediatric age group. Echocardiography should be done early in children presenting with ischaemic thromboembolic diseases in order to reduce morbidity and mortality rates resulting from cardiac pathology.
